# Supplementary figures and images for: Improved base editing and functional screening in Leishmania via co-expression of the AsCas12a ultra variant, a T7 RNA polymerase, and a cytosine base editor
Source: eLife. 2025 Feb 24;13:RP97437. doi: 10.7554/eLife.97437 (PMC11850003; doi:10.7554/eLife.97437)

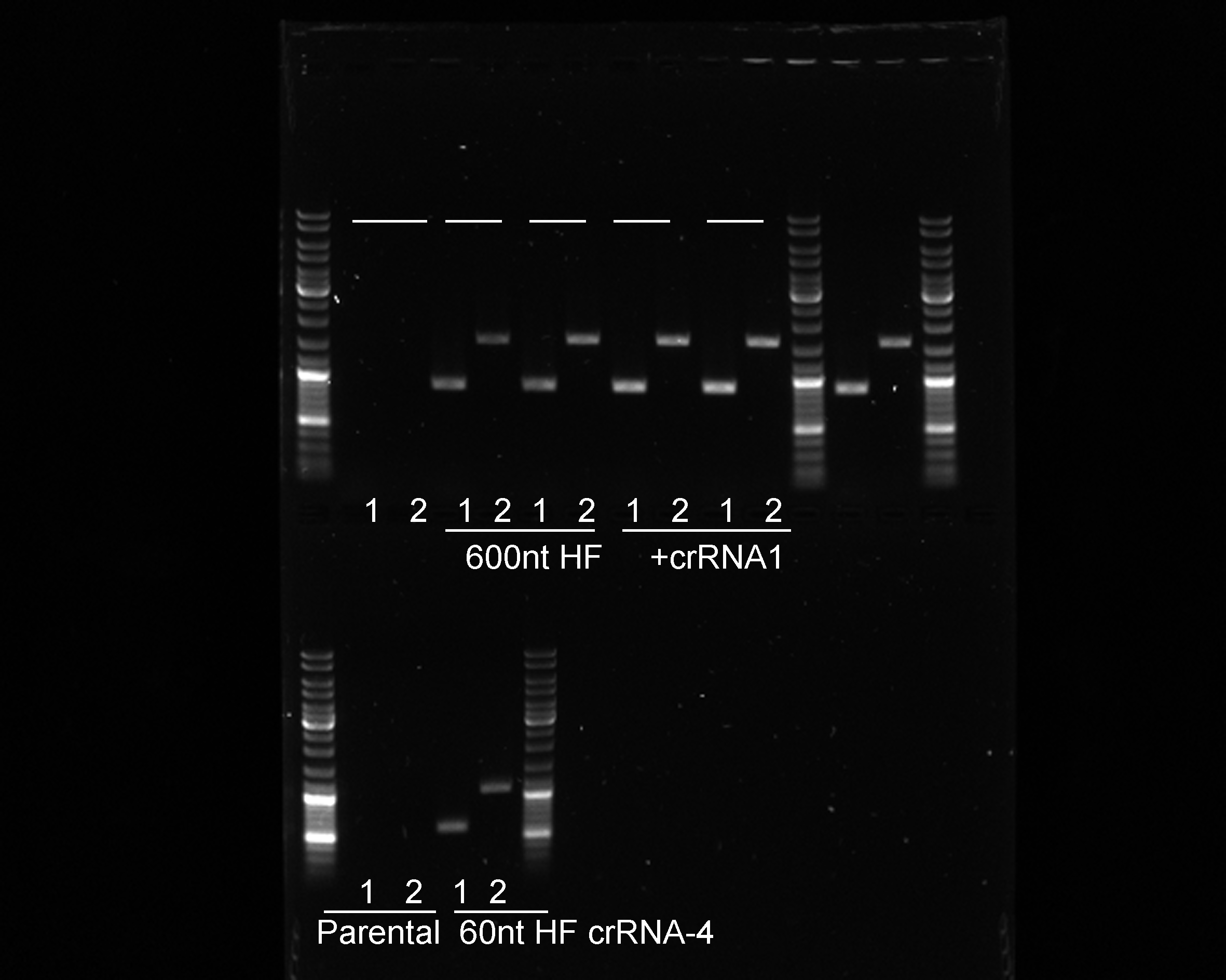

Supplement: Figure 2—figure supplement 1—source data 1. [file elife-97437-fig2-figsupp1-data1.zip › Source data 1 - Raw DNA images of Figure 2 S1B and E (labelled).tif]

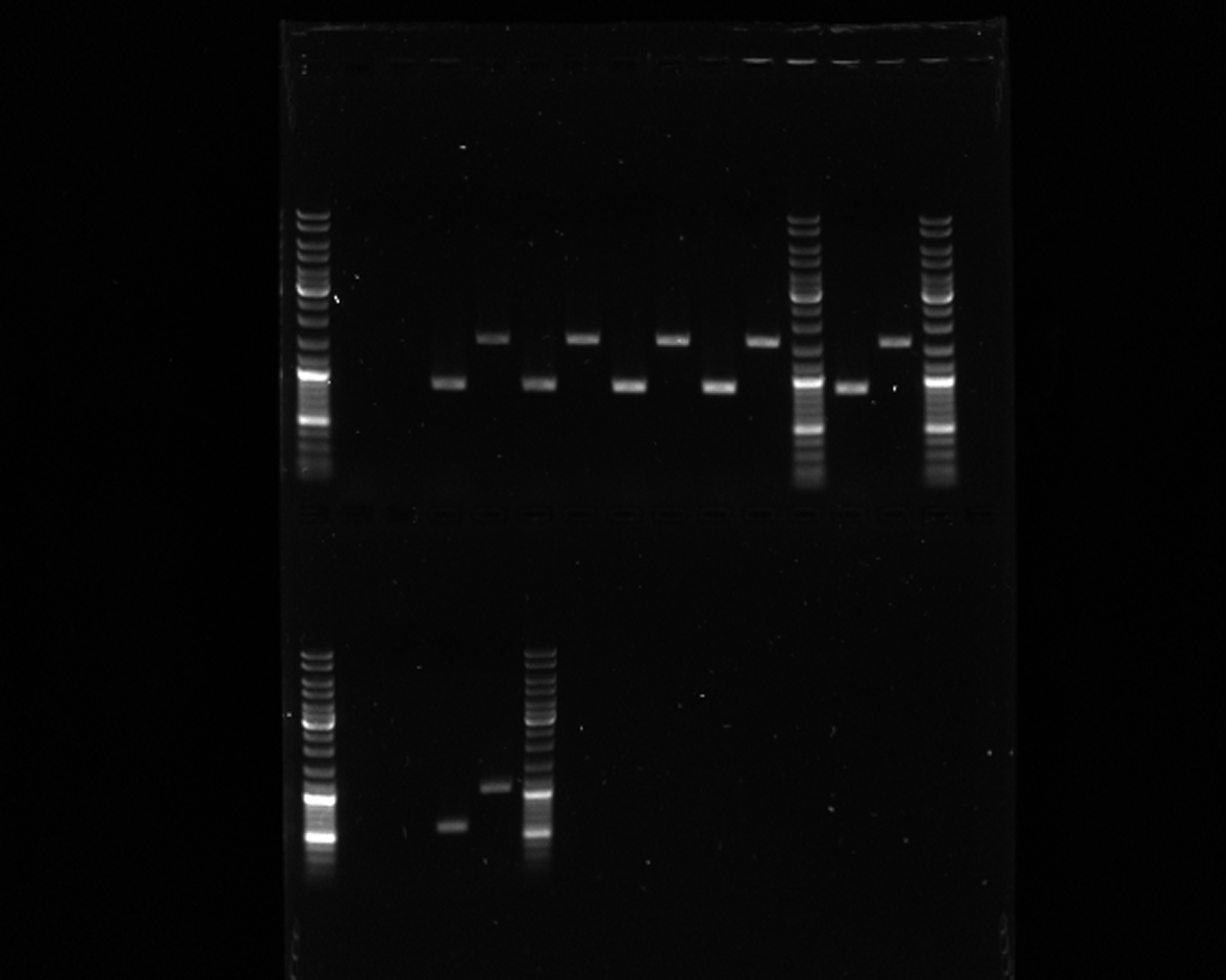

Supplement: Figure 2—figure supplement 1—source data 2. [file elife-97437-fig2-figsupp1-data2.zip › Source data 1 - Raw DNA images of Figure 2 S1B and E.tif]

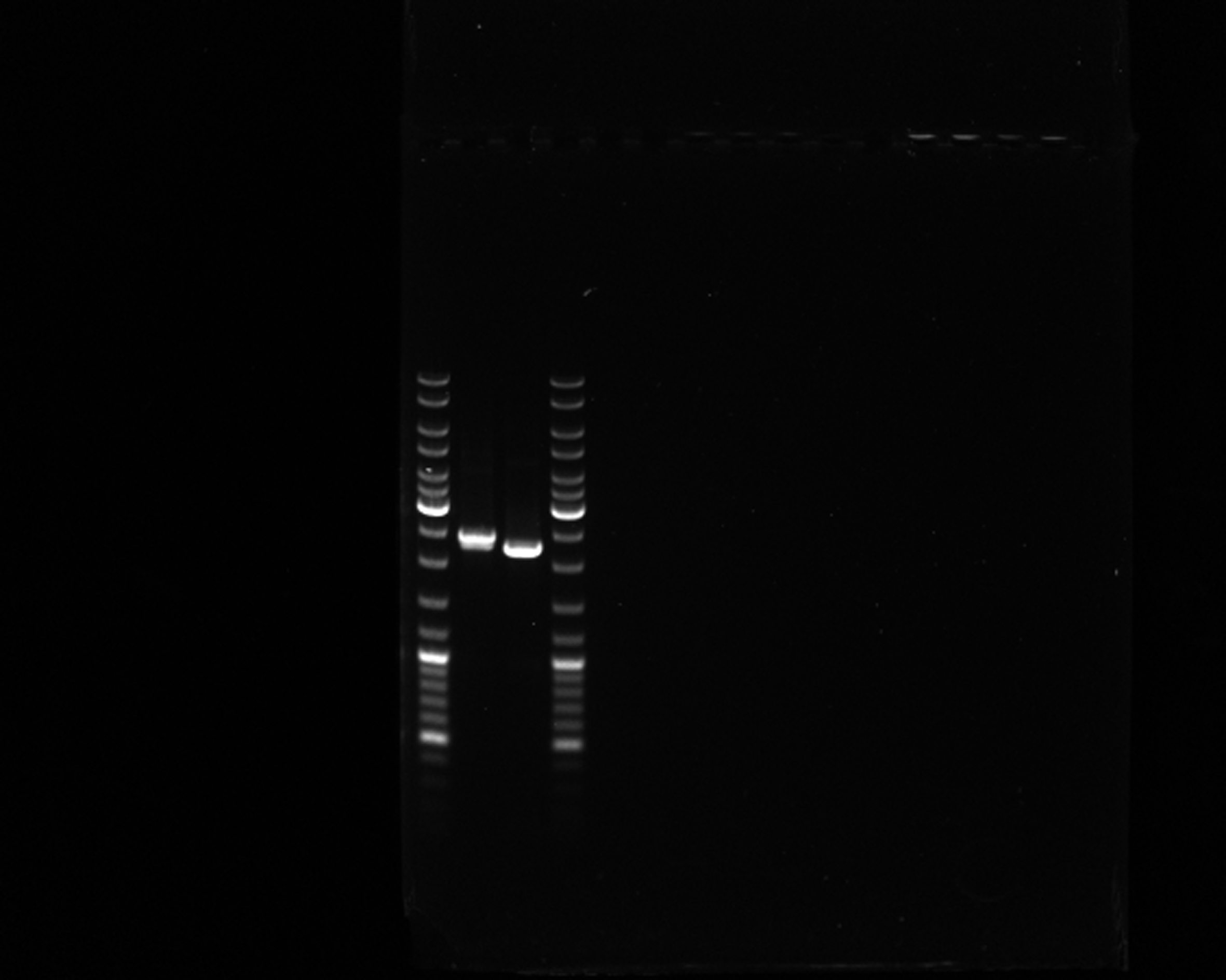

Supplement: Figure 6—source data 1. [file elife-97437-fig6-data1.zip › Source data 2 - Raw DNA images of Figure 6B (labelled).tif]

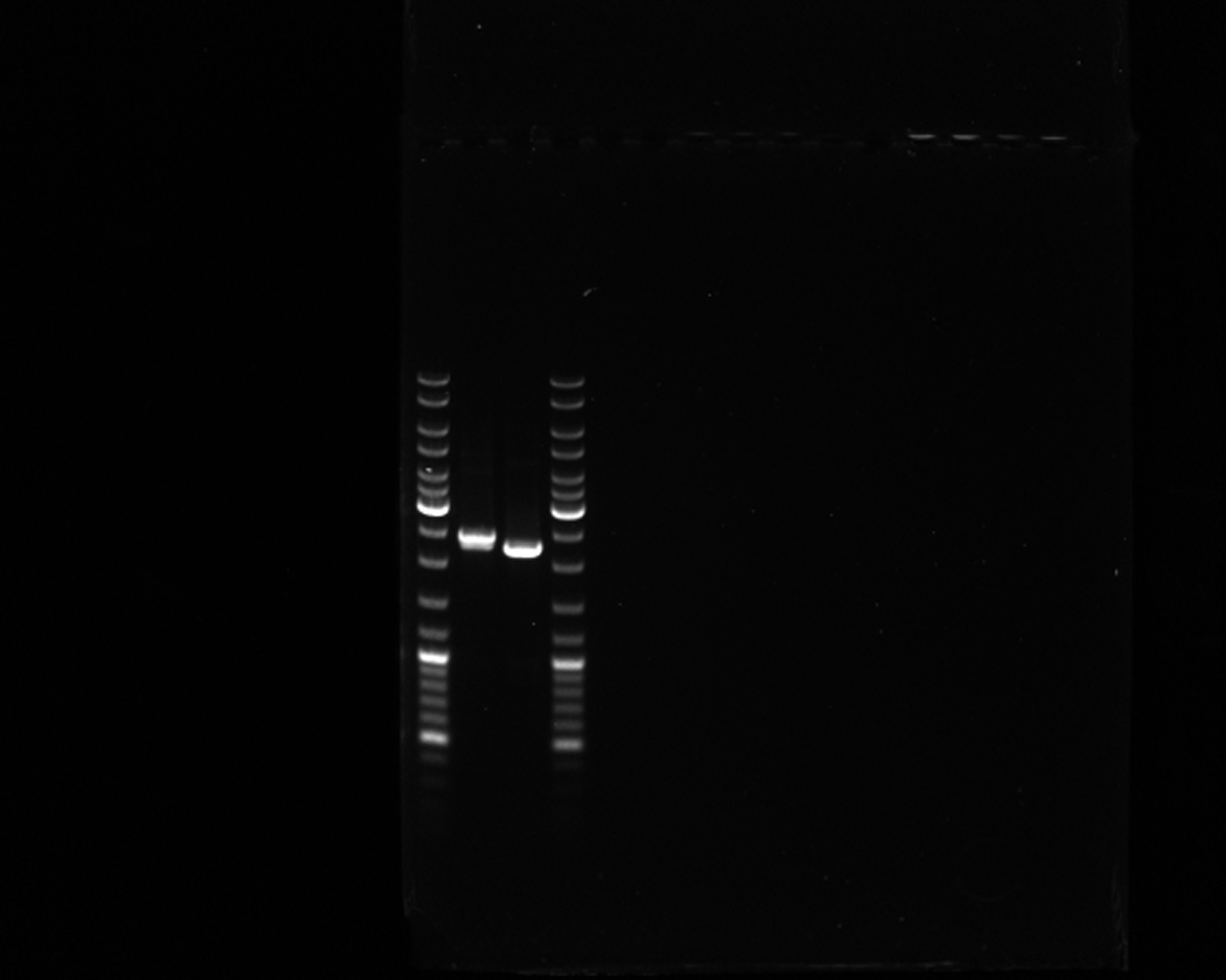

Supplement: Figure 6—source data 2. [file elife-97437-fig6-data2.zip › Source data 2 - Raw DNA images of Figure 6B.tif]
